# Resveratrol ameliorated endothelial injury of thoracic aorta in diabetic mice and Gly‐LDL‐induced HUVECs through inhibiting TLR4/HIF‐1α

**DOI:** 10.1111/jcmm.16584

**Published:** 2021-06-10

**Authors:** Wenjun Sha, Meizhi Liu, Dusang Sun, Junhui Qiu, Bilin Xu, Lin Chen, Tian Shen, Cheng Chen, Hongping Wang, Cuiping Zhang, Tao Lei

**Affiliations:** ^1^ Department of Endocrinology Putuo Hospital Shanghai University of Traditional Chinese Medicine Shanghai China

**Keywords:** resveratrol, thoracic aorta, TLR4/HIF‐1α

## Abstract

To explore the effects of resveratrol on the levels of inflammatory cytokines and Toll‐like receptor‐4/ hypoxia‐inducible transcription factors‐1α (TLR4/HIF‐1α) signalling pathway in diabetes mellitus. C57BL/6 mice received intraperitoneal injection of streptozocin for constructing diabetic mice models. Human umbilical vein endothelial cells (HUVECs) were treated with 50 µg/mL Gly‐LDL for inducing injury models. 10, 100 and 1000 mmol/L resveratrol were obtained and added into each group. Haematoxylin‐eosin (H&E) staining was used for histological evaluation. CCK8 assay was performed for determination of cell viability, and Transwell assay was implemented for detecting cell migration ability. Cell apoptosis was analysed using flow cytometry. The content of inflammatory factors including interleukin‐6 (IL‐6), tumour necrosis factor‐α (TNF‐α), vascular adhesion molecule‐1 (VCAM‐1) and vascular endothelial growth factor (VEGF) were measured by ELISA. GST pull‐down assay was employed for determining interactions between TLR4 and HIF‐1α. The protein expression of TLR4 and HIF‐1α was detected using Western blotting and immunohistochemistry, while relative mRNA expression was measured by RT‐qRCR. Resveratrol could reduce bodyweight and ameliorate endothelial injury of thoracic aorta in diabetic mice. Both in vivo and in vitro results revealed that the level of IL‐6, TNF‐α, VCAM‐1 and VEGF was significantly down‐regulated after being treated with resveratrol. Resveratrol inhibited the increase of MDA and ROS and increased the level of SOD in diabetic mice. Western blotting, IHC and RT‐qPCR results showed that the levels of TLR4 and HIF‐1α were significantly down‐regulated in resveratrol group. Overexpression of TLR4 or HIF‐1α could reverse the effect of resveratrol. GST pull‐down elucidated that there might be a close interaction between TLR4 and HIF‐1α. Resveratrol ameliorated endothelial injury of thoracic aorta in diabetic mice and Gly‐LDL‐induced HUVECs through inhibiting TLR4/HIF‐1α signalling pathway.

## 1 INTRODUCTION

Diabetes mellitus (DM), a metabolic disease, contributes to a variety of complications, such as vascular disease resulting from inflammation.^1^, ^2^ Recent data have revealed that the population of DM has increased over the years. It has been reported that 451 million people will suffer from diabetes in 2017 and the number will increase to 693 million by 2045.^3^ However, there is presently no dramatic illustration on preventing the occurrence of DM. And the majority of patients are more liable to die from macrovascular complications,^4^ such as cardiovascular disease, hypertension and stroke. The outcomes of these abnormalities do cause immense suffering and place a heavy burden on health care. Thus, it is urgent and critical to explore a novel mechanism for treating DM clinically.

Endothelial dysfunction commonly occurs in the early stage of cardiovascular disease, which is accompanied by inflammatory responses.^5^ Toll‐like receptor‐4 (TLR4), as a critical inflammatory mediator, plays a crucial role in maintaining the function of vascular endothelium.^6^ Inflammatory cytokines such as vascular adhesion molecule (VCAM)‐1, vascular endothelial growth factor (VEGF), tumour necrosis factor‐α (TNF‐α) and interleukin‐6 (IL‐6), results in various oxidant enzymes synthesis and tissue damage and activate a serious of inflammation.^7^, ^8^ As the downstream factors of TLR4, those mediators have received considerable attention, and the possibilities of mechanism studies have been fascinating. Recently, inflammation has been identified as a process resulting in aortic expansion^9^; thus, the underlying mechanism of inflammatory cytokines need to be further revealed. In addition, hypoxia‐inducible transcription factors (HIFs) have an important role in adapting to various oxygenation states.^10^ HIF‐1 is a principal mediator of adapting to low oxygen and is typically determined under low oxygen levels.^11^ Researchers have shown that HIF is imperative for vascular development, as the absence of HIF‐1α will damage vascular system injury, causing early embryonic lethality.^12^ However, the functional mechanism of HIF‐1α along with TLR4 regulating vascular endothelia remains unclear. LDL uptake is related to the modification of lipid and lipid stores of cells.^13^ Patients suffering from DM are frequently associated with lipid abnormalities, which contribute to the unstainable metabolism of glycated low‐density lipoprotein (Gly‐LDL), one of the most novel fields in lipoprotein metabolism throughout the years.^14^ More research highlighted that long‐term hyperglycaemia could accelerate the oxidation of LDL, being related to the formation of atherosclerotic lesions.^15^ Bowie et al found that atherosclerosis in DM might be correlated to Gly‐LDL via its increased susceptibility to oxidation.^16^ In this way, the role of Gly‐LDL further dominates the deterioration of vascular diseases.^17^

Resveratrol, a polyphenolic phytoalexin, can be commonly found in a variety of plants and products, such as grapes, red wine, barriers and peanut skins,^18^ showing some unique advantages over anti‐oxidative, anti‐ageing and anti‐cancer effects.^19^ Apart from this, it cannot be ignored by its hypoglycaemic anti‐inflammatory action. Chalons et al found that resveratrol exhibits a critical effect on inducing apoptosis and anti‐tumour and disturbing the cell cycle of cancer cells.^20^ Cai et al illustrated that resveratrol could improve glucose homeostasis and possess potential anti‐inflammatory properties.^21^ However, it cannot conclude to clarify the mechanism of resveratrol exerting beneficial on vascular endothelial injury induced by DM. In the current study, the effects of resveratrol on the levels of inflammatory cytokines were explored and the potential mechanism of TLR4/HIF‐1α signalling pathway was studied, which offers a new spectrum of clinical treatment in DM.

## 2 METHODS

### 2.1 Animal group

A total of 50 male C57BL/6 mice (6‐7 weeks old) were obtained from National Rodent Laboratory Animal Resources Shanghai Branch. The mice were housed at room temperature under a controlled 12 hours light and 12 hours dark cycle and had free access to water and food, receiving humane care according to the criteria outlined in the ‘Guide for the Care and Use of Laboratory Animals’. The mice were divided into 5 groups (n = 10 in each group): Control group: the mice received intraperitoneal injection of citrate buffer. Model group: the mice received intraperitoneal injection of streptozocin (STZ) (Sigma‐Aldrich, St. Louis, MO, USA) at a dose of 50 mg/kg bodyweight for 5 consecutive days. After two weeks of treatment, a glucometer was implemented for detecting glucose of mice blood that was collected via mandibular vein puncture. A fasting‐blood glucose higher then 12 mmol/L was considered diabetic mice which were used for subsequent experiments. Model + 10 mmol/L resveratrol (V900386, C_14_H_12_O_3_, molecular weight 228.24, purity ≥98%, Sigma‐Aldrich) group: diabetic mice were fed a diet enriched with 10 mmol/L resveratrol for 16 weeks. Model + 100 mmol/L resveratrol group: diabetic mice were fed a diet enriched with 100 mmol/L resveratrol for 16 weeks. Model + 1 mol/L resveratrol group: diabetic mice were fed a diet enriched with 1 mol/L resveratrol for 16 weeks. Then, the final bodyweight was recorded and the blood samples were collected, after which the mice were killed using carbon dioxide asphyxiation.

The overexpression vector of TLR4 and HIF‐1α was constructed by HanBio Co. Ltd., Shanghai, China, via lentivirus expressing system. The lent‐OE‐TLR4 as well as lent‐OE‐HIF‐1α was transfected into mice via tail vein injection, while lent‐OE‐NC was used as control. A total of 60 male C57BL/6 mice were divided into 6 groups (n = 10 in each group). The mice in model group received intraperitoneal injection of streptozocin (STZ) at a dose of 50 mg/kg bodyweight for 5 consecutive days. For resveratrol treatment group, the diabetic mice were fed a diet enriched with 1 mol/L resveratrol for 16 weeks. The divided groups were: Model group, Model + 1 mol/L resveratrol group, Model + 1 mol/L resveratrol group + OE‐TLR4 group, Model + 1 mol/L resveratrol group + OE‐NC1 group, Model + 1 mol/L resveratrol group + OE‐ HIF‐1α group and Model + 1 mol/L resveratrol group + OE‐NC2 group. After 18 weeks, the final bodyweight was recorded and the blood samples were collected, after which the mice were killed using carbon dioxide asphyxiation. All animal studies were reviewed and approved by Putuo Hospital, Shanghai University of Traditional Chinese Medicine.

### 2.2 Cell culture

Human umbilical vein endothelial cells (HUVECs) were obtained (Lonza, Beijing, China) and cultured in Dulbecco's modified Eagle medium containing 10% foetal bovine serum according to the standard protocols. Cells were divided into 5 groups: Control group: cells were treated with PBS solution. Model group: cells were treated with 50 µg/mL Gly‐LDL. Model + 10 mmol/L resveratrol group: cells were treated with 50 µg/mL Gly‐LDL and 10 mmol/L resveratrol. Model + 100 mmol/L resveratrol group: cells were treated with 50 µg/mL Gly‐LDL and 100 mmol/L resveratrol. Model + 1 mol/L resveratrol group: cells were treated with 50 µg/mL Gly‐LDL and 1 mol/L resveratrol. The HUVECs were cultured in a 5% CO_2_ humidity incubator at 37°C. The medium was replaced every two days. When the confluence of cells reached 80%‐90%, the cells were extracted for subsequent experiments.

For construction of in vitro TLR4 and HIF‐1α expression models, lent‐OE‐TLR4 and lent‐OE‐HIF‐1α (HanBio, Shanghai, China) was transfected into HUVECs using Lipofectamine 2000 (Invitrogen, Waltham, MA, USA) according to the manufacturer's protocols. The cells were divided into 6 groups: Model group, Model + 1 mol/L resveratrol group, Model + 1 mol/L resveratrol group + OE‐TLR4 group, Model + 1 mol/L resveratrol group + OE‐NC1 group, Model + 1 mol/L resveratrol group + OE‐ HIF‐1α group and Model + 1 mol/L resveratrol group + OE‐NC2 group. HUVECs were incubated at 37°C for 24 hours and then used for subsequent experiments.

### 2.3 Histological evaluation

Thoracic aorta tissues of mice were extracted and fixed in 10% buffered formalin solution. After incubating at room temperature for 30 minutes, tissues were dehydrated in 75% ethanol overnight and then embedded in paraffin. The specimens were cut into 5‐μm‐thick serial sections, and haematoxylin‐eosin (H&E) staining was performed for evaluation of histological changes. Results of H&E staining were observed by an optical microscope.

### 2.4 Immunohistochemistry

Immunohistochemistry (IHC) was conducted according to the manufacture's instructions. Paraffin‐embedded 2‐μm‐thick tissues were dewaxed in xylene and rehydrated in graded alcohol, after which 5% goat serum was added for blocking and the tissues were incubated with primary antibody at 4°C overnight. The primary antibodies included TLR4 (ab22048, 1:500, Abcam, Cambridge, USA) and HIF‐1α (ab51608, 1:500, Abcam). The quantification of TLR4 and HIF‐1α expression was measured by two independent pathologists. The IHC scores were determined by the combination of the percentage of positive staining and the staining intensity.

### 2.5 CCK8 assay

The cells in logarithmic growth phase were digested with trypsin to prepare cell suspension at the density of 5 × 10^4^ cells/mL. Then, cells were inoculated into 96‐well plates (100 μL per well) and then cultured in an incubator with 5% CO2 at 37℃ for 12 hours. Then, 10 μL Cell Counting Kit‐8 (CCK‐8) solution obtained from Keygen Biotech, Nanjing, China, was added into each well and incubated for 2 hours as per the instructions. The absorbance of each well was detected at 450 nm wavelength.

### 2.6 Transwell assay

Transwell assay was performed as per the manufacturer's instructions. Cells were dispensed onto the upper well of the transwell chamber and precoated with 0.5% gelatin. Totally, 450 μL DEME that contained 20% FBS was filled into the lower transwell chamber. The plates were incubated at 37°C in a humidified incubator with 5% CO_2_ for 24 hours. After removing non‐migrated cells, migrated cells were stained using crystal violet die. Migrated cells were observed and recorded using a microscope.

### 2.7 ELISA analysis

The content of IL‐6, TNF‐α, VCAM‐1 and VEGF were determined by ELISA (Bioscience, San Diego, CA, USA) according to the manufacture's manual.

### 2.8 Measurement of oxidative status

Biochemical assay kits (Nanjing Jiangcheng Bioengineering Institute, Nanjing, China) were used for detection of malondialdehyde (MDA), superoxide dismutase (SOD) and reactive oxygen species (ROS) according to the manufacture's protocol.

### 2.9 GST pull‐down assay

GST pull‐down assay was implemented to identify the interactions between TLR4 and HIF‐1α. For obtaining purified proteins, sequences encoding TLR4 were cloned into vector pEGX‐6P‐1 that contained open reading frame of GST tag, and sequences encoding HIF‐1α were cloned into vector pET22b that contained open reading frame of 6 × His tag. All the vectors were expressed in *Escherichia coli* BL21 and purified. Then, glutathione‐sepharose beads (Amersham Pharmacia Biotech, Shanghai, China) were incubated with purified GST‐fused proteins in a rotating incubator at 4°C for 12 hours. Then, the beads were collected and washed for 3 times, after which input proteins were incubated with the beads in a rotating incubator at 4°C for 3 hours. The supernatant was removed and beads were collected and washed, and target proteins were eluted and detected using Western blotting.

### 2.10 Immunofluorescence staining

Thoracic aorta tissues of mice were fixed with 4% polyformaldehyde for 20 minutes. Samples were treated with 0.5% Triton X‐100 and blocked with 10% FBS for 1 hours at room temperature. Anti‐HIF‐1α (ab179483, 1:500, Abcam) and anti‐TLR4 (ab22048, 1:500, Abcam) were added and incubated overnight at 4°C. Subsequently, DyLight 594‐labelled goat anti‐mouse IgG (1:40) and DyLight 488‐labelled goat anti‐rabbit IgG (1:30) were added and incubated at 37°C for 2 hours. DAPI was used for nuclei staining (C1002, Beyotime Institute of Biotechnology). A fluorescent microscope was implemented of observation.

### 2.11 Western blotting

Thoracic aorta tissues of mice or cells were collected and lysed with radio immunoprecipitation assay buffer that supplemented with protease inhibitor and phosphatase inhibitor, after which the proteins were quantified. After proteins being separated by SDS‐polyacrylamide gel electrophoresis (SDS‐PAGE), samples were transferred to polyvinylidene difluoride (PVDF) membranes (Millipore, Massachusetts, USA). 5% nonfat milk solution was used for blocking and incubated for 1 hour at room temperature, after which primary antibody including caspase‐3 (ab13847, 1:500, Abcam), BAX (ab32503, 1:500, Abcam), BCL‐2 (ab32124, 1:500, Abcam), TLR4 (ab22048, 1:500, Abcam) and HIF‐1α (ab179483, 1:500, Abcam) were added and incubated for 12 hours at 4°C. Then, samples were reacted with HRP‐conjugated secondary antibody for 1.5 hours at room temperature. The bands on the membranes were observed using a chemiluminescence analyser obtained from Bio‐Rad, CA, USA.

### 2.12 RT‐qPCR analysis

The total RNA was extracted from tissues or cells using TRIzol reagent. First‐strand cDNA was synthesized using RevertAidTM First Strand cDNA Synthesis Kit according to the manufacturer's instructions with the following condition: 16°C for 30 minutes, 37°C for 30 minutes, and 70°C for 10 minutes. The RT‐qPCR analysis was performed on CFX96 Real‐Time PCR Detection System with SYBR Premix Ex TaqTM (TaKaRa, Dalian, China). The relative expression of mRNA was calculated by the formula of 2^−ΔΔCt^ method. The primer sequences were list as follow:

TLR4‐F: TTCTGCAATGTCTCTGGCAGG;

TLR4‐R: GCTGAGACTTGGTAGGGCCA;

HIF‐1a‐F: ATCCATGTGACCATGAGGAAATG;

HIF‐1a‐R: CCGGCTTGTTAGGGTGCACTTC;

GAPDH‐F: AGGTCGGTGTGAACGGATTTG;

GAPDH‐R: TGTAGACCATGTAGTTGAGGTCA.

### 2.13 Flow cytometry analysis

The cells were added digested with trypsin and incubated at room temperature for 1 hour. Then, the trypsin was removed and the cells were centrifuged at 1000 *g* for 5 minutes. The supernatant was discarded and the cells were collected, after which the cells were resuspended with PBS and centrifuged again at 1000 *g* for 5 minutes. The supernatant was discarded and 220 μL of annexin V‐FITC solution was added, after which 15 μL of propidium iodide solution was added and incubated at room temperature for 20 minutes. Cells washed with PBS were resuspended in medium supplemented with 0.5% bovine serum albumin and detected by flow cytometry.

### 2.14 Statistical analysis

SPSS 24.0 was implemented to conduct all statistical analysis. All experiments were performed in triplicates, and all statistical data were expressed as ±standard deviation (SD). The comparisons among multiple groups were evaluated by one‐way analysis of variance (ANOVA). LSD test was performed for pairwise comparison when the variance was homogeneous; otherwise, Kruskal–Wallis h test was used. *P* < .05 was considered statistically significant.

## 3 RESULTS

### 3.1 Resveratrol ameliorated endothelial injury of thoracic aorta and inflammation status in vivo

**FIGURE 1:**
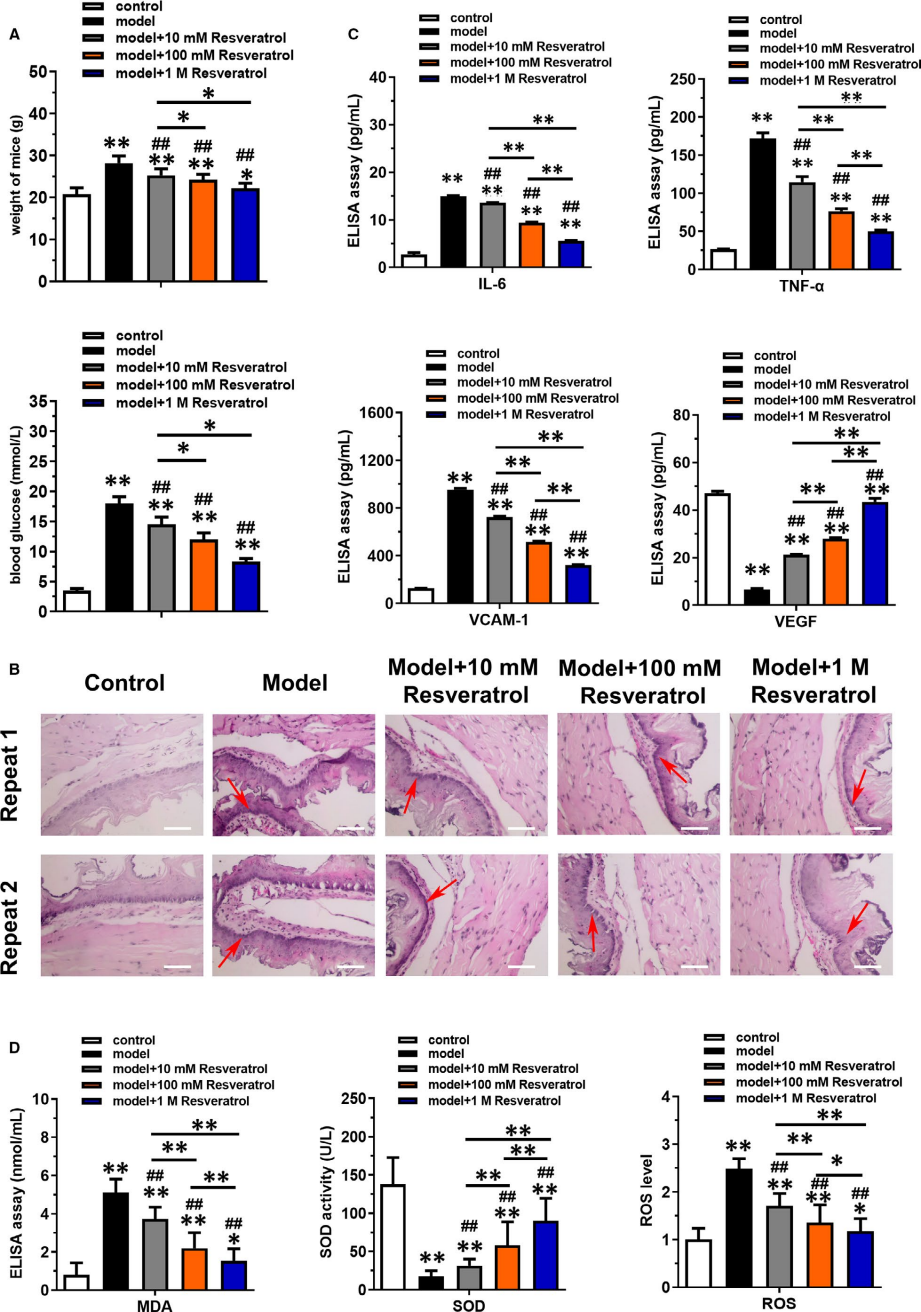
Resveratrol ameliorated endothelial injury of thoracic aorta in diabetic mice and reduced inflammatory factor secretion and oxidative stress in a dose‐dependent manner. The diabetic mice model constructed by the injection of STZ was purchased from SHANGHAI SLAC LABORATORY ANIMAL CO. and treated with different doses of resveratrol (10, 100 mmol/L, 1 mol/L). The normal mice were served as control group. A, Bodyweight and blood glucose of the mice in each group were detected to determine the success of the diabetic model. B, H&E staining was used to detect the injury of thoracic aorta. Scale bar = 50 μm. C, ELISAs were used to detect the expression of inflammatory factors (IL‐6, TNF‐α, VCAM‐1) and VEGF in serum. D, Commercial kits were employed to detect oxidative stress factors including MDA, SOD and ROS. *P < .05 and **P < .01 compared with control group, ^#^ *P* < .05, ^##^ *P* < .01 compared with model group

The diabetic mice model was constructed by the injection of STZ for evaluation the effect of resveratrol. As presented in Figure 1A, the weight and blood glucose of mice were significantly increased in model group compared with control group, which was significantly reversed by the resveratrol in a dose‐dependent manner (*P* < .05). However, the 2 and 4 mol/L of resveratrol treatment exhibited no significant differences on the weight and blood glucose of mice treated with 1 mol/L resveratrol (Figure S1). H&E staining results showed in Figure 1B illustrated that resveratrol could dramatically alleviate the endothelial injury of thoracic aorta in diabetic mice. ELISAs were implemented to detect the expression of inflammatory factors that included IL‐6, TNF‐α, VCAM‐1 and VEGF in serum (Figure 1C). Results indicated that the levels of IL‐6, TNF‐α and VCAM‐1 were up‐regulated and VEGF was down‐regulated in model group, while resveratrol could reverse the effects in a dose‐dependent manner (*P* < .05). Oxidative stress factors including MDA, SOD and ROS were also detected using commercial kits (Figure 1D), which results revealed that resveratrol inhibited the increase of MDA and ROS and increased the level of SOD in model group (*P* < .05).

### 3.2 Resveratrol inhibited the expression of TLR4 and HIF‐1α

**FIGURE 2:**
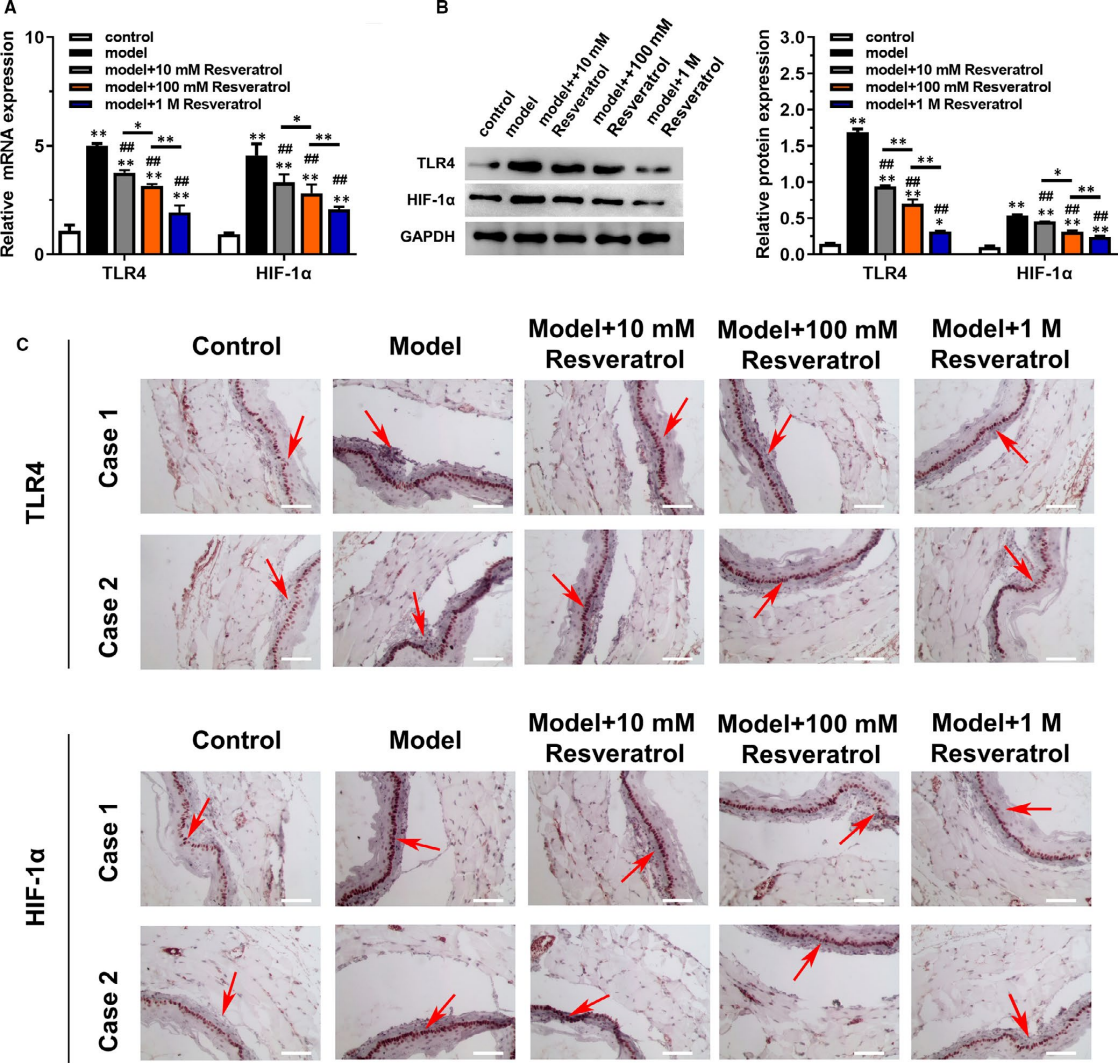
Resveratrol inhibited the expression of TLR4 and HIF‐1α in thoracic aorta of diabetic mice in a dose‐dependent manner. The diabetic mice model was treated with different doses of resveratrol (10, 100 mmol/L, 1 mol/L). A, The expression of relative mRNA of TLR4 and HIF‐1α in each group was detected by RT‐qPCR. B, The expression of relative protein of TLR4 and HIF‐1α in each group was detected by Western blotting. C, IHC staining was used to detect the expression of TLR4 and HIF‐1α in thoracic aorta. Scale bar = 50 μm. *P < .05 and **P < .01 compared with control group, ^#^ *P* < .05, ^##^ *P* < .01 compared with model group

RT‐qPCR was employed for measuring mRNA expression of TLR4 and HIF‐1α. Results in Figure 2A showed that the levels of TLR4 and HIF‐1α were significantly increased in model group, while resveratrol decreased these levels in a dose‐dependent manner (*P* < .05). Consistently, Western blotting analysis and IHC results further confirmed the up‐regulation of expression of TLR4 and HIF‐1α in model group, and resveratrol could down‐regulate this expression (Figure 2B,C).

### 3.3 Overexpression of TLR4 or HIF‐1α reversed the effect of resveratrol in diabetic mice

**FIGURE 3:**
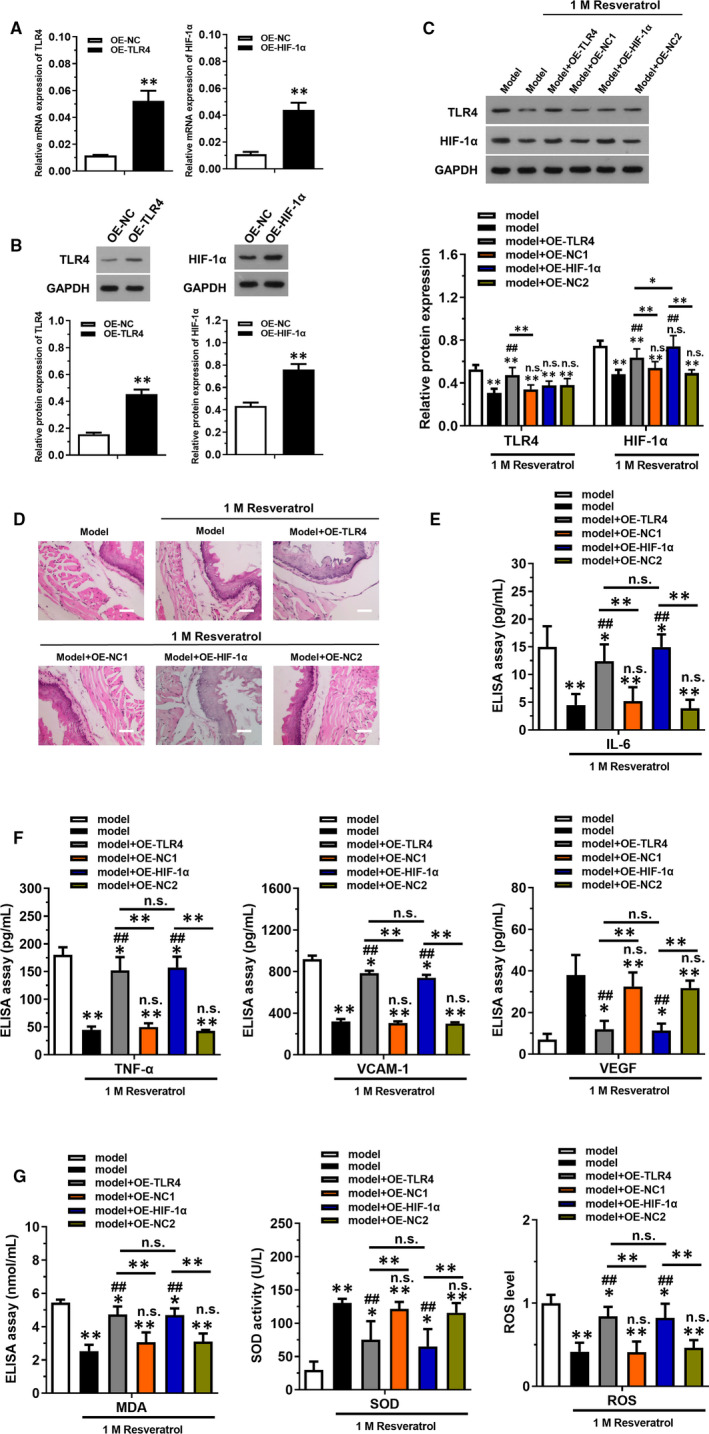
Overexpression of TLR4 or HIF‐1 α reversed the effect of resveratrol on thoracic aortic injury in diabetic mice. The diabetic mice model was treated with 1 mol/L resveratrol. Lentivirus plasmids were injected into caudal vein to overexpress TLR4 (lent‐OE‐TLR4) and HIF‐1α (lent‐OE‐HIF‐1α) stably. (A and B) The transfection efficiency of lent‐OE‐TLR4 and lent‐OE‐HIF‐1α was detected using RT‐qPCR and Western blotting. C, The relative protein expression of TLR4 and HIF‐1α in each group was examined by Western blotting. D, H&E staining was used to detect the injury of thoracic aorta. Scale bar = 50 μm. (E and F) ELISAs were used to detect the expression of inflammatory factors (IL‐6, TNF‐α, VCAM‐1) and VEGF in serum. (G) Commercial kits were employed to detect oxidative stress factors including MDA, SOD and ROS. *P < .05 and **P < .01 compared with model group, ^#^ *P* < .05, ^##^ *P* < .01 compared with model group with 1 mol/L resveratrol treatment, NS represented no significance

In vivo overexpression model of TLR4 and HIF‐1α was constructed, respectively, for elucidating the mechanism of resveratrol. RT‐qPCR was implemented to detect the relative mRNA expression in OE‐TLR4 (Figure 3A) and OE‐HIF‐1α (Figure 3B) group. Data showed that the levels of TLR4 and HIF‐1α were significantly up‐regulated in the overexpression group compared with OE‐NC group, indicating that the models were successfully constructed (*P* < .05). Western blotting was used to determine the protein expression in each overexpression group (Figure 3C). Results showed that only HIF‐1α were up‐regulated in OE‐HIF‐1α group, while overexpression of TLR4 could up‐regulate both TLR4 and HIF‐1α expression compared with model + 1 mol/L resveratrol group (*P* < .05). H&E staining results revealed that overexpression of TLR4 and HIF‐1α could reverse the effect of 1 mol/L resveratrol on thoracic aortic injury (Figure 3D). Inflammatory factors included IL‐6, TNF‐α, VCAM‐1 and VEGF in serum (Figure 3E,F) was also increased in OE‐TLR4 and OE‐HIF‐1α group compared with model + 1 mol/L resveratrol group (*P* < .05). Finally, the levels of MDA and ROS were up‐regulated while that of SOD was down‐regulated in OE‐TLR4 and OE‐HIF‐1α group compared with model + 1 mol/L resveratrol group (*P* < .05).

### 3.4 TLR4 interacted with HIF‐1α

**FIGURE 4:**
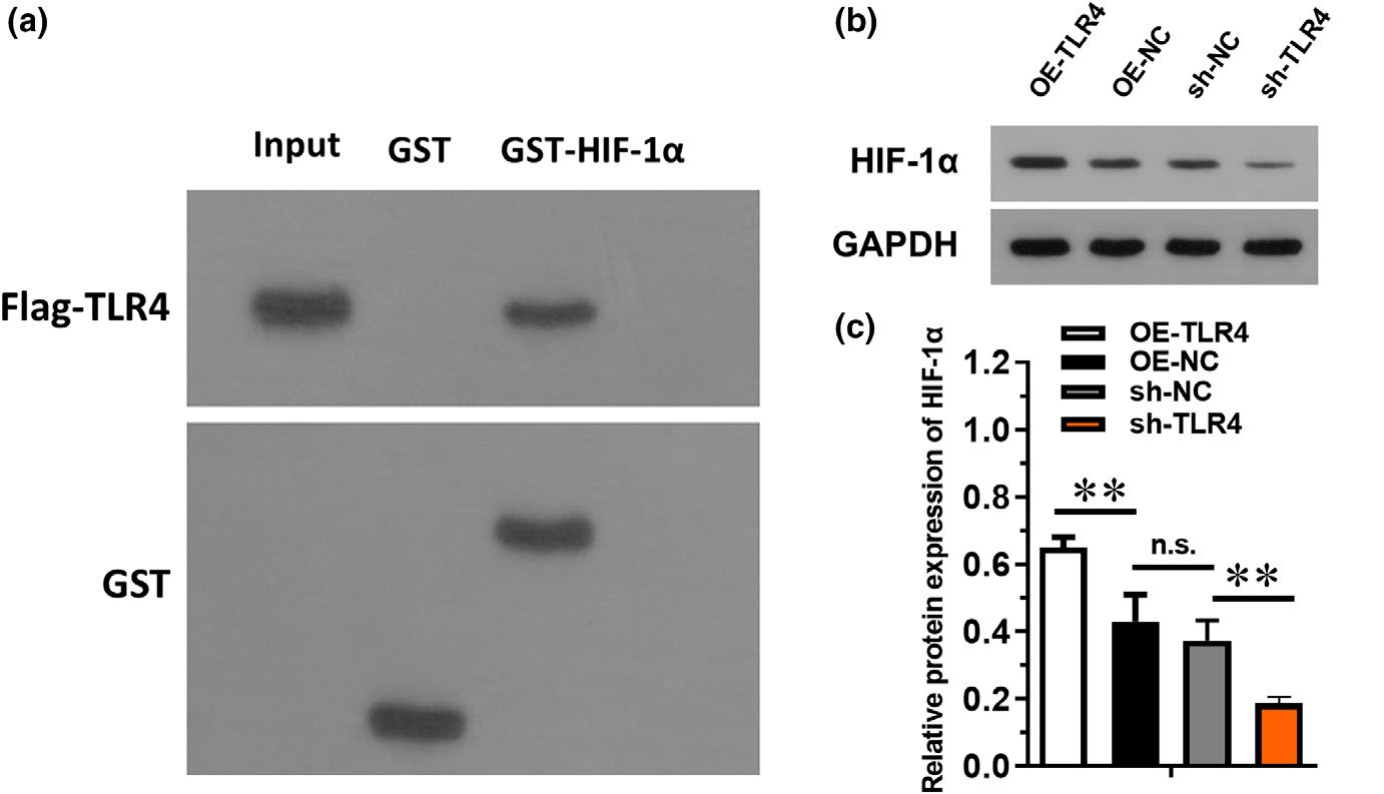
The regulation of TLR4 on HIF‐1α. A, The protein binding of TLR4 to HIF‐1α was detected by GST pull‐down method. B, The interaction between the protein expression of TLR4 and HIF‐1α was examined using Western blotting. C, The interaction between TLR4 and HIF‐1α determined by colocalization. Green dots represent HIF‐1α expression; red dots represent TLR4 expression; blue dots represent nucleus. * or ** represented *P* < .05 or *P* < .01, NS represented no significance

To investigate the mechanism of TLR4 on modulating injury and inflammatory status in diabetic mice, GST pull‐down assays were implemented to explore the interaction between TLR4 and HIF‐1α (Figure 4A). Results revealed that GST‐tagged TLR4 protein could pull‐down His‐tagged HIF‐1α protein. Moreover, Western blotting was performed, and the data demonstrated that overexpression of TLR4 could significantly up‐regulate HIF‐1α expression compared with OE‐NC group (Figure 4B), while inhibiting TLR4 using sh‐TLR4 would dramatically decrease HIF‐1α level compared with sh‐NC group (*P* < .05). Moreover, colocalization of TLR4 and HIF‐1α detected by immunofluorescence staining further demonstrated our hypothesis that TLR4 might co‐expressed with HIF‐1α (Figure 4C). These evidences suggested that there might be a close interaction between TLR4 and HIF‐1α.

### 3.5 Resveratrol ameliorated Gly‐LDL‐induced damage in HUVECs

**FIGURE 5:**
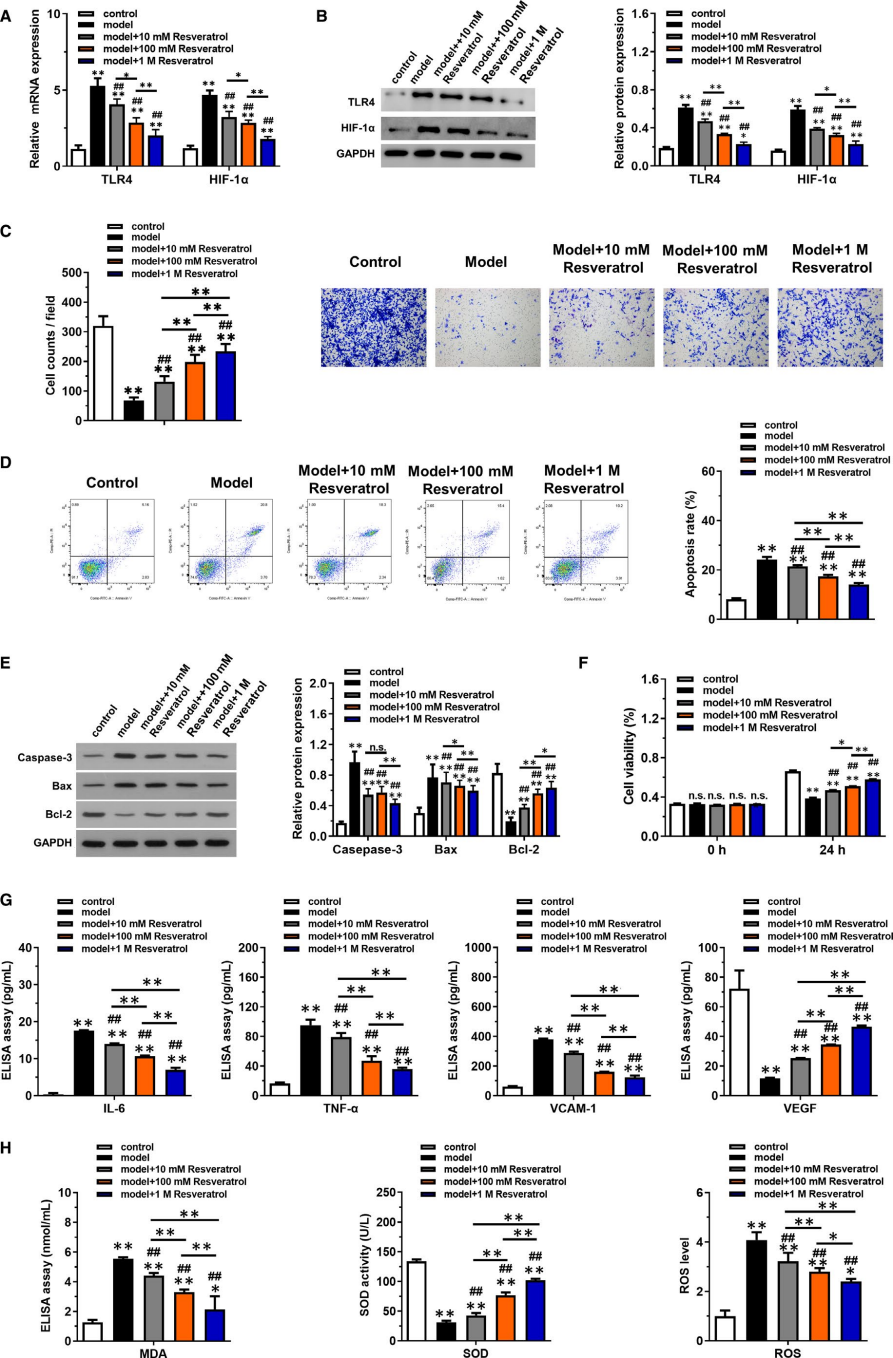
Resveratrol promoted cell migration, inhibited Gly‐LDL‐induced apoptosis and inflammatory factors and oxidative stress in a dose‐dependent manner. HUVECs were treated with Gly‐LDL to induce the injury model. The cell model was treated with different doses of resveratrol (10, 100 mmol/L, 1 mol/L). A, The expression of relative mRNA of TLR4 and HIF‐1α in each group was detected by RT‐qPCR. B, The expression of relative protein of TLR4 and HIF‐1α in each group was detected by Western blotting. C, Cell migration in groups with indicated treatments was detected using Transwell method. D, Cell apoptosis in groups with indicated treatments was detected by flow cytometry. E, The expression of apoptotic proteins including caspase 3, Bax and Bcl‐2 was examined using Western blotting. F, Cell viability in groups with indicated treatments was detected by CCK‐8 kits. G, ELISAs were used to detect the expression of inflammatory factors (IL‐6, TNF‐α, VCAM‐1) and VEGF in serum. H, Commercial kits were employed to detect oxidative stress factors including MDA, SOD and ROS. *P < .05 and **P < .01 compared with control group, ^#^ *P* < .05 and ^##^ *P* < .01 compared with model group, NS represented no significance

In vitro experiments were executed for further confirming the findings. HUVECs were treated with Gly‐LDL to induce the injury model. Consistent with in vivo experiments, the relative mRNA levels of TLR4 and HIF‐1α were significantly increased in model group (Figure 5A), while treatment of resveratrol could decrease their expression in a dose‐dependent manner (*P* < .05). Protein levels in each group detected using Western blotting further confirmed the change of mRNA level (Figure 5B). Cell migration ability was detected using Transwell assay, which results showed that resveratrol promoted cell migration that originally inhibited by Gly‐LDL in a dose‐dependent manner (Figure 5C). Then, cell apoptosis was measured by flow cytometry analysis (Figure 5D). It could be concluded that Gly‐LDL increased cell apoptotic rate of HUVECs, while resveratrol had the reversed effects (*P* < .05). The apoptotic proteins including caspase‐3, Bax and Bcl‐2 were also examined (Figure 5E), which results showed that resveratrol could significantly down‐regulate caspase‐3 and Bax expression and up‐regulate Bcl‐2 expression (*P* < .05). Moreover, cell ability at 24 hours was increased in resveratrol group compared with model group (Figure 5F). The Gly‐LDL‐induced up‐regulation of inflammatory factors IL‐6, TNF‐α, VCAM‐1 and VEGF in serum were also down‐regulated by resveratrol in a dose‐dependent manner (Figure 5G). Finally, oxidative stress factors including MDA, SOD and ROS were detected (Figure 5H), which results indicated that resveratrol decreased MDA and ROS level while increased SOD level compared with model group (*P* < .05).

### 3.6 Overexpression of TLR4 or HIF‐1α reversed the effect of resveratrol on Gly‐LDL‐treated HUVECs

**FIGURE 6:**
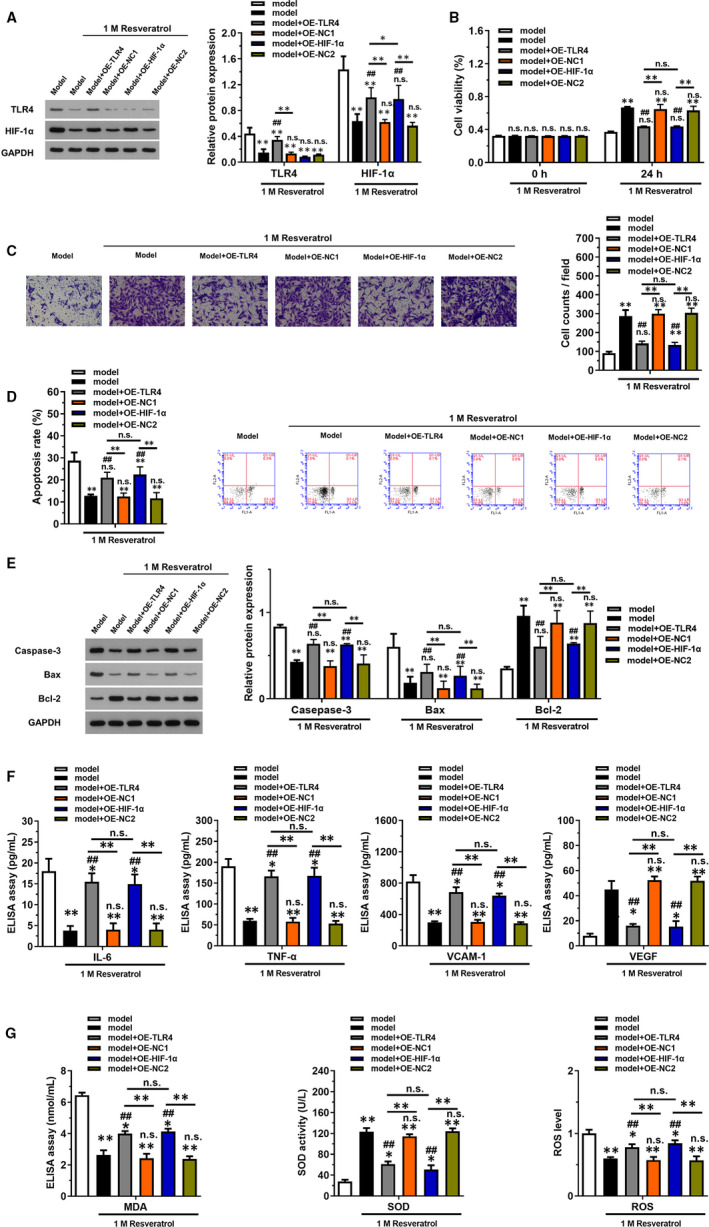
Overexpression of TLR4 or HIF‐1 α reversed the effect of resveratrol on Gly‐LDL‐induced thoracic aortic injury in HUVECs. Lentivirus plasmids were transfected into HUVECs to overexpress TLR4 (lent‐OE‐TLR4) and HIF‐1α (lent‐OE‐HIF‐1α) stably. The cells were treated with 1 mol/L resveratrol. A, The relative protein expression of TLR4 and HIF‐1α in each group was examined by Western blotting. B, Cell viability in groups with indicated treatments was detected by CCK‐8 kits. C, Cell migration in groups with indicated treatments was detected using Transwell method. D, Cell apoptosis in groups with indicated treatments was detected by flow cytometry. E, The expression of apoptotic proteins including caspase 3, Bax and Bcl‐2 was examined using Western blotting. F, ELISAs were used to detect the expression of inflammatory factors (IL‐6, TNF‐α, VCAM‐1) and VEGF in serum. G, Commercial kits were employed to detect oxidative stress factors including MDA, SOD and ROS. *P < .05 and **P < .01 compared with model group, ^#^ *P* < .05, ^##^ *P* < .01 compared with model group with 1 mol/L resveratrol treatment, NS represented no significance

In vitro overexpression model of TLR4 and HIF‐1α was constructed, respectively, for elucidating the mechanism of resveratrol. Western blotting results showed that protein expression of TLR4 and HIF‐1α were both increased in OE‐TLR4 group, while only HIF‐1α was up‐regulated in OE‐ HIF‐1α group (Figure 6A). Cell viability detected using CCK‐8 assay (Figure 6B) revealed that overexpression of TLR4 and HIF‐1α significantly decreased cell viability at 24 hours compared with model + resveratrol group (*P* < .05). Furthermore, cell migration ability (Figure 6C) was also inhibited in OE‐TLR4 and OE‐ HIF‐1α group compared with model + resveratrol group (*P* < .05). Flow cytometry results (Figure 6D) and the change of apoptotic protein levels (Figure 6E) revealed that overexpression of TLR4 and HIF‐1α promoted cell apoptosis via down‐regulating Bcl‐2 expression and up‐regulating caspase‐3 and Bax expression. The levels of inflammatory factors (Figure 6F) were up‐regulated in OE‐TLR4 and OE‐ HIF‐1α group compared with model + resveratrol group (*P* < .05), suggesting that overexpression of TLR4 and HIF‐1α promoted the inflammatory status in HUVECs. Moreover, the oxidative status (Figure 6G) was also promoted by overexpression of TLR4 and HIF‐1α through increasing MDA and ROS levels and decreasing SOD level (*P* < .05).

## 4 DISCUSSION

Diabetes mellitus and its complications have contributed tremendously to the burden of regions experiencing economic and medical issues.^22^ Accumulated evidence has also suggested that diabetes‐associated cardiovascular complications have difficulty in treating effectively.^23^ It is thus possible to establish novel approaches for chronic inflammation induced by DM to prevent the adverse effect of vascular complications.

Resveratrol has been verified to be beneficial to metabolic effects and insulin sensitivity.^24^ And research has confirmed that resveratrol acts against inflammatory disease in mice.^25^ Tung et al showed that resveratrol could lessen the effect of TNF‐α and IL‐1β, indicating it has anti‐inflammatory activity.^26^ Accordingly, the hypoglycaemic effect of resveratrol has been extending in the therapy of diabetes and cardiovascular complications.

The occurrence of diabetes is strongly linked to inflammation, which stimulates diabetes‐related cardiovascular disease.^27^ Accumulated evidence suggested that resveratrol and its metabolites contribute to an inhibitory regulation of the inflammatory cascade in endothelial cells and the secretion of pro‐inflammatory factors, such as VEGF and IL‐8, decrease in TNF‐α‐activated cells.^28^ Hou et al found that High levels of plasma angiotensin Ⅱ, a symbol of insulin resistance, could be reversed by resveratrol.^29^ Li et al illustrated that resveratrol could also regulate immune cell function, prevent the immune cell infiltration into the vascular wall and improve the function of surrounding adipose tissue, which protects the function of vascular.^30^ A strong relationship between pro‐inflammatory cytokines and postprandial blood glucose has been reported in the previous study.^31^ Zhang et al suggested that the inhibitor of Sirt1, a target molecular of resveratrol, hindered the inhibitory effect of resveratrol on TLR4.^32^ As the key factor of TLR4, IL‐6 plays a critical role in the activation of macrophages, which impacts glucose homeostasis and limits inflammation.^33^ In addition, VEGF is closely related to vascular inflammation and VCAM‐1 released by vascular endothelium is deemed to an activator of atherosclerosis.^34^ However, the underlying mechanism between resveratrol and vascular endothelial injury is elusive.

In the current study, we further investigated the effect of resveratrol on Gly‐LDL‐induced endothelial damage of thoracic aorta in DM rats. The results showed that resveratrol alleviated the endothelial injury of the thoracic aorta and decreased inflammatory secretion in a dose‐dependent. The previous study evaluating the effect on resveratrol observed in DM rats have noted that with the treatment of resveratrol, the cardiovascular function was improved via inhibiting VEGF and suppressing p38 phosphorylation.^35^ According to the analysis of PCR, Western blotting and IHC, resveratrol could suppress the expression of TLR4 and HIF‐1α, while their overexpression could reverse the effect of resveratrol in DM rats with thoracic aortic tissue injury, indicating that vascular endothelial injury in DM rats might be associated with the expression inflammatory cytokines regulated by resveratrol. These results are in line with those of previous studies. Manesh et al suggested that islet cytokine secretomes were altered via TLR4, having impacted its downstream inflammasome.^36^ Besides, down‐regulating the expression of HIF‐1α, which was dramatically increased in DM rats, could reverse hyperglycaemia damages such as vascular dysfunction and occlusion.^37^ In the vitro experiment, resveratrol restrained the apoptosis and facilitated migration in HUVECs reduced by Gly‐LDL in a dose‐dependent. The expression of inflammatory factors and the level of oxidative stress in HUVECs were accordingly suppressed. Furthermore, the result of overexpressed TLR4 and HIF‐1α was the same as vivo experiment, reversing the effect of resveratrol on inhibiting Gly‐LDL‐induced endothelial dysfunction. Prior studies were designed to determine the interaction between TLR4 and HIF‐1α. Yang et al ^38^ illustrated that TLR4 was co‐expressed with HIF‐1α via activating the lipid rafts/NADPH oxidase redox signalling. Kim et al^39^ found HIF‐1α could up‐regulate TLR4 in macrophages to response to hypoxic stress. Although we cannot conclude to have demonstrated the specific mechanism by which TLR4 and HIF‐1α interact, our studies strongly suggest that their close link on the levels of inflammatory cytokines and DM, thus this warrants further investigation.

In conclusion, our research showed the effect of resveratrol on improving the levels of inflammatory cytokines via TLR4/HIF‐1α signalling pathway in DM rats and revealed the possible approaches to improving endothelial damage. However, the interaction effect among resveratrol, Gly‐LDL and inflammatory cytokines was not considered as much as possible, which is a potential limitation. The comparison of the protective effects among similar polyphenols that included catechins would also be explored in the future. Therefore, further research should be undertaken to investigate the interaction among a certain specific component and signalling pathways.

## CONFLICT OF INTEREST

The authors declare that they have no competing interests.

## AUTHOR CONTRIBUTIONS

**Wenjun Sha:** Conceptualization (equal); Data curation (equal); Formal analysis (equal); Investigation (equal); Methodology (equal); Resources (equal); Supervision (equal); Validation (equal); Visualization (equal); Writing‐original draft (equal); Writing‐review & editing (equal). **Meizhi Liu:** Conceptualization (equal); Data curation (equal); Formal analysis (equal); Investigation (equal); Methodology (equal); Resources (equal); Supervision (equal); Validation (equal); Visualization (equal); Writing‐original draft (equal); Writing‐review & editing (equal). **Dusang Sun:** Data curation (equal); Formal analysis (equal); Investigation (equal); Methodology (equal); Resources (equal); Software (equal); Supervision (equal); Validation (equal); Visualization (equal). **Junhui Qiu:** Data curation (equal); Formal analysis (equal); Methodology (equal); Resources (equal); Supervision (equal); Validation (equal). **Bilin Xu:** Data curation (equal); Formal analysis (equal); Investigation (equal); Methodology (equal); Resources (equal); Supervision (equal); Validation (equal). **Lin Chen:** Data curation (equal); Formal analysis (equal); Methodology (equal); Resources (equal); Supervision (equal); Visualization (equal). **Tian Shen:** Data curation (equal); Formal analysis (equal); Methodology (equal); Validation (equal); Visualization (equal). **Cheng Chen:** Data curation (equal); Formal analysis (equal); Investigation (equal); Supervision (equal); Validation (equal). **Hongping Wang:** Formal analysis (equal); Investigation (equal); Software (equal); Supervision (equal); Validation (equal). **Cuiping Zhang:** Formal analysis (equal); Methodology (equal); Software (equal); Validation (equal). **Tao Lei:** Funding acquisition (equal); Software (equal); Supervision (equal); Visualization (equal).

## ETHICAL APPROVAL AND CONSENT TO PARTICIPATE

The research protocol was reviewed and approved by the Ethical Committee and Institutional Review Board of the Putuo Hospital, Shanghai University of Traditional Chinese Medicine.

## CONSENT TO PUBLISH

All of the authors have consented to publish this research.

## Supporting information

Fig S1

## Data Availability

The data that support the findings of this study are available from the corresponding author upon reasonable request.

## References

[1] Duh E , Aiello LP . Vascular endothelial growth factor and diabetes: the agonist versus antagonist paradox. Diabetes. 1999;48(10):1899‐1906. 10512352 10.2337/diabetes.48.10.1899

[2] Onat D , Brillon D , Colombo PC , et al. Human vascular endothelial cells: a model system for studying vascular inflammation in diabetes and atherosclerosis. Curr Diab Rep. 2011;11(3):193‐202. 21337131 10.1007/s11892-011-0182-2 PMC3311155

[3] Cho NH , Shaw JE , Karuranga S , et al. IDF Diabetes Atlas: global estimates of diabetes prevalence for 2017 and projections for 2045. Diabetes Res Clin Pract. 2018;138:271‐281. 29496507 10.1016/j.diabres.2018.02.023

[4] van den Born JC , Hammes H‐P , Greffrath W , et al. Gasotransmitters in vascular complications of diabetes. Diabetes. 2016;65(2):331‐345. 26798119 10.2337/db15-1003

[5] Rocha DM , Caldas AP , Oliveira LL , et al. Saturated fatty acids trigger TLR4‐mediated inflammatory response. Atherosclerosis. 2016;244:211‐215. 26687466 10.1016/j.atherosclerosis.2015.11.015

[6] Harrington LS , Belcher E , Moreno L , et al. Homeostatic role of Toll‐like receptor 4 in the endothelium and heart. J Cardiovasc Pharmacol Ther. 2007;12(4):322‐326. 18172227 10.1177/1074248407306217

[7] Zhu T , et al. Andrographolide protects against LPS‐induced acute lung injury by inactivation of NF‐κB. PLoS One. 2013;8(2):e56407. 23437127 10.1371/journal.pone.0056407 PMC3578846

[8] Cheng S‐C , Wu Y‐H , Huang W‐C , et al. Anti‐inflammatory property of quercetin through downregulation of ICAM‐1 and MMP‐9 in TNF‐α‐activated retinal pigment epithelial cells. Cytokine. 2019;116:48‐60. 30685603 10.1016/j.cyto.2019.01.001

[9] Shah PK . Inflammation, metalloproteinases, and increased proteolysis: an emerging pathophysiological paradigm in aortic aneurysm. Circulation. 1997;96(7):2115‐2117. 9337176 10.1161/01.cir.96.7.2115

[10] Palazon A , Tyrakis PA , Macias D , et al. An HIF‐1α/VEGF‐A axis in cytotoxic T cells regulates tumor progression. Cancer Cell. 2017;32(5):669‐683.e5. 29136509 10.1016/j.ccell.2017.10.003 PMC5691891

[11] Cramer T , Yamanishi Y , Clausen BE , et al. HIF‐1alpha is essential for myeloid cell‐mediated inflammation. Cell. 2003;112(5):645‐657. 12628185 10.1016/s0092-8674(03)00154-5 PMC4480774

[12] Gerri C , Marín‐Juez R , Marass M , Marks A , Maischein HM , Stainier DYR . Hif‐1α regulates macrophage‐endothelial interactions during blood vessel development in zebrafish. Nat Commun. 2017;8:15492. 28524872 10.1038/ncomms15492 PMC5493593

[13] Khosravi M , Hosseini‐Fard R , Najafi M . Circulating low density lipoprotein (LDL). Horm Mol Biol Clin Investig. 2018;35(2). 10.1515/hmbci-2018-0024 30059347

[14] van den Boogert MAW , Rader DJ , Holleboom AG . New insights into the role of glycosylation in lipoprotein metabolism. Curr Opin Lipidol. 2017;28(6):502‐506. 28922188 10.1097/MOL.0000000000000461

[15] Sobal G , Menzel EJ , Sinzinger H . Calcium antagonists as inhibitors of in vitro low density lipoprotein oxidation and glycation. Biochem Pharmacol. 2001;61(3):373‐379. 11172743 10.1016/s0006-2952(00)00548-7

[16] Bowie A , Owens D , Collins P , Johnson A , Tomkin GH . Glycosylated low density lipoprotein is more sensitive to oxidation: implications for the diabetic patient? Atherosclerosis. 1993;102(1):63‐67. 8257453 10.1016/0021-9150(93)90084-8

[17] Lee Y‐T , Hsu C‐C , Lin M‐H , et al. Histidine and carnosine delay diabetic deterioration in mice and protect human low density lipoprotein against oxidation and glycation. Eur J Pharmacol. 2005;513(1–2):145‐150. 15878720 10.1016/j.ejphar.2005.02.010

[18] Rauf A , Imran M , Butt MS , et al. Resveratrol as an anti‐cancer agent: a review. Crit Rev Food Sci Nutr. 2018;58(9):1428‐1447. 28001084 10.1080/10408398.2016.1263597

[19] Carter LG , D'Orazio JA , Pearson KJ . Resveratrol and cancer: focus on in vivo evidence. Endocr Relat Cancer. 2014;21(3):R209‐R225. 24500760 10.1530/ERC-13-0171 PMC4013237

[20] Chalons P , Amor S , Courtaut F , et al. Study of potential anti‐inflammatory effects of red wine extract and resveratrol through a modulation of interleukin‐1‐beta in macrophages. Nutrients. 2018;10(12):1856. 10.3390/nu10121856 PMC6316397 30513737

[21] Cai TT , Chen H , Wang YY , et al. Resveratrol modulates the gut microbiota and inflammation to protect against diabetic nephropathy in mice. Front Pharmacol. 2020;11:1249. 32973502 10.3389/fphar.2020.01249 PMC7466761

[22] Zheng Y , Ley SH , Hu FB . Global aetiology and epidemiology of type 2 diabetes mellitus and its complications. Nat Rev Endocrinol. 2018;14(2):88‐98. 29219149 10.1038/nrendo.2017.151

[23] Naito R , Miyauchi K . Coronary Artery Disease and Type 2 Diabetes Mellitus. Int Heart J. 2017;58(4):475‐480. 28717115 10.1536/ihj.17-191

[24] Springer M , Moco S . Resveratrol and its human metabolites‐effects on metabolic health and obesity. Nutrients. 2019;11(1):143. 10.3390/nu11010143 PMC6357128 30641865

[25] Sun H , Cai H , Fu Y , et al. The protection effect of resveratrol against radiation‐induced inflammatory bowel disease via NLRP‐3 inflammasome repression in mice. Dose Response. 2020;18(2):1559325820931292. 32636719 10.1177/1559325820931292 PMC7323307

[26] Tung BT , Rodríguez‐Bies E , Talero E , et al. Anti‐inflammatory effect of resveratrol in old mice liver. Exp Gerontol. 2015;64:1‐7. 25687021 10.1016/j.exger.2015.02.004

[27] Breuss JM , Atanasov AG , Uhrin P . Resveratrol and its effects on the vascular system. Int J Mol Sci. 2019;20(7):1523. 10.3390/ijms20071523 PMC6479680 30934670

[28] Toaldo IM , Van Camp J , Gonzales GB , et al. Resveratrol improves TNF‐α‐induced endothelial dysfunction in a coculture model of a Caco‐2 with an endothelial cell line. J Nutr Biochem. 2016;36:21‐30. 27560195 10.1016/j.jnutbio.2016.07.007

[29] Hou C‐Y , Tain Y‐L , Yu H‐R , et al. The effects of resveratrol in the treatment of metabolic syndrome. Int J Mol Sci. 2019;20(3):535. 10.3390/ijms20030535 PMC6387422 30695995

[30] Li H , Xia N , Hasselwander S , Daiber A . Resveratrol and vascular function. Int J Mol Sci. 2019;20(9):2155. 10.3390/ijms20092155 PMC6539341 31052341

[31] Ohishi M . Hypertension with diabetes mellitus: physiology and pathology. Hypertens Res. 2018;41(6):389‐393. 29556093 10.1038/s41440-018-0034-4

[32] Zhang M , Xue Y , Chen H , et al. Resveratrol inhibits MMP3 and MMP9 expression and secretion by suppressing TLR4/NF‐κB/STAT3 activation in Ox‐LDL‐Treated HUVECs. Oxid Med Cell Longev. 2019;2019:9013169. 31583048 10.1155/2019/9013169 PMC6754947

[33] Pedersen BK . Anti‐inflammatory effects of exercise: role in diabetes and cardiovascular disease. Eur J Clin Invest. 2017;47(8):600‐611. 28722106 10.1111/eci.12781

[34] Fan M , Bai J , Ding T , et al. Adipose‐derived stem cell transplantation inhibits vascular inflammatory responses and endothelial dysfunction in rats with atherosclerosis. Yonsei Med J. 2019;60(11):1036‐1044. 31637885 10.3349/ymj.2019.60.11.1036 PMC6813142

[35] Yan F , Sun X , Xu C . Protective effects of resveratrol improve cardiovascular function in rats with diabetes. Exp Ther Med. 2018;15(2):1728‐1734. 29434758 10.3892/etm.2017.5537 PMC5774431

[36] Chittezhath M , Wai CMM , Tay VSY , et al. TLR4 signals through islet macrophages to alter cytokine secretion during diabetes. J Endocrinol. 2020;247(1):87. 32755994 10.1530/JOE-20-0131

[37] Maugeri G , D'Amico AG , Saccone S , et al. PACAP and VIP inhibit HIF‐1α‐mediated VEGF expression in a model of diabetic macular edema. J Cell Physiol. 2017;232(5):1209‐1215. 27661459 10.1002/jcp.25616

[38] Yang X , Chen GT , Wang YQ , et al. TLR4 promotes the expression of HIF‐1α by triggering reactive oxygen species in cervical cancer cells in vitro‐implications for therapeutic intervention. Mol Med Rep. 2018;17(2):2229‐2238. 29207048 10.3892/mmr.2017.8108 PMC5783462

[39] Kim SY , Choi YJ , Joung SM , et al. Hypoxic stress up‐regulates the expression of Toll‐like receptor 4 in macrophages via hypoxia‐inducible factor. Immunology. 2010;129(4):516‐524. 20002786 10.1111/j.1365-2567.2009.03203.x PMC2842498

