# Supplementary figures and images for: Resveratrol ameliorated endothelial injury of thoracic aorta in diabetic mice and Gly‐LDL‐induced HUVECs through inhibiting TLR4/HIF‐1α
Source: J Cell Mol Med. 2021 Jun 10;25(13):6258–70. doi: 10.1111/jcmm.16584 (PMC8256346; doi:10.1111/jcmm.16584)

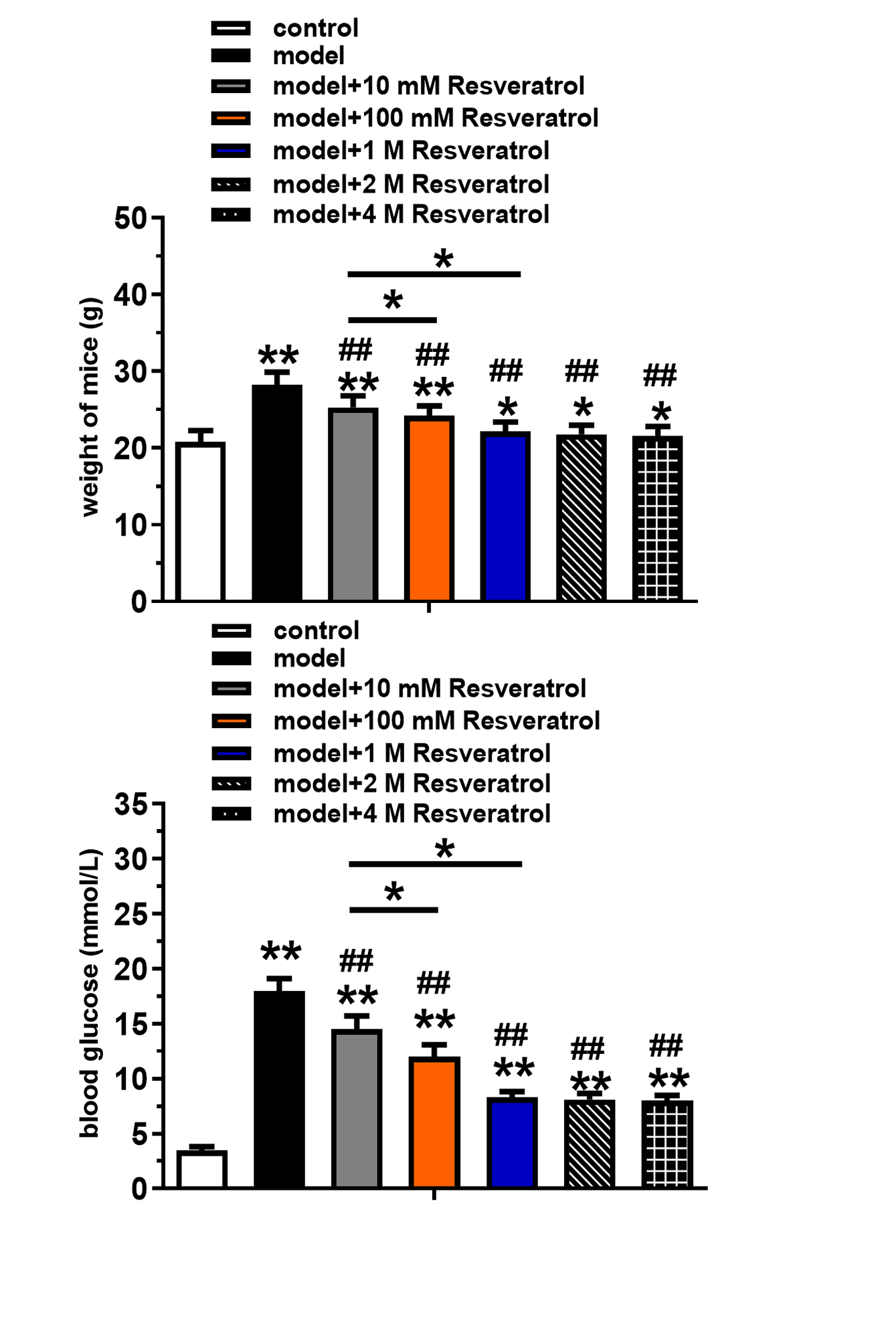

Supplement: Supplementary file 1 — Fig S1 [file JCMM-25-6258-s001.tif]
